# Editorial: Data Based Radiation Oncology—Design of Clinical Trials

**DOI:** 10.3389/fonc.2018.00034

**Published:** 2018-02-16

**Authors:** Kerstin Anne Kessel, Anne W. M. Lee, Søren M. Bentzen, Bhadrasain Vikram, Fridtjof Nüsslin, Stephanie E. Combs

**Affiliations:** ^1^Department of Radiation Oncology, Klinikum rechts der Isar, Technische Universität München, Munich, Germany; ^2^Institute for Innovative Radiotherapy (iRT), Helmholtz Zentrum München, Munich, Germany; ^3^Department of Clinical Oncology, The University of Hong Kong Shenzhen Hospital, Shenzhen, China; ^4^Division of Biostatistics and Bioinformatics, Department of Epidemiology and Public Health, The Greenebaum Cancer Center, School of Medicine, University of Maryland, Baltimore, MD, United States; ^5^National Cancer Institute (NIH), Rockville, MD, United States

**Keywords:** clinical trials, data collection, radiation oncology, clinical study design, study management

**Editorial on the Research Topic**

**Data Based Radiation Oncology—Design of Clinical Trials**

In radiation oncology as in many other specialties, clinical trials are essential to investigate new therapeutic approaches. Usually, preparation for a prospective clinical trial is time-consuming until ethics approval is obtained. To test a new treatment many years pass before it can be implemented in the routine care. During that time, already new interventions emerge, new drugs appear on the market, technical and physical innovations are being implemented, novel biology-driven concepts are translated into clinical approaches while we are still investigating the ones from years ago.

Another problem is associated with molecular diagnostics and the growing amount of tumor-specific biomarkers which allow for better stratification of patient subgroups. On the other side, this may result in a much longer time for patient recruiting and consequently in larger multicenter trials. Moreover, all of the relevant data must be readily available for treatment decision making, treatment as well as follow-up, and ultimately for trial evaluation. This challenges even more for agreed standards in data acquisition, quality, and management.

How could we change the way currently clinical trials are performed in a way they are safe and ethically justifiable and speed up the initiation process so that we can provide new and better treatments faster for our patients?

Furthermore, while we rely on various quantitative information handling distributed, large heterogeneous amounts of data efficiently is very important. Thus, data management becomes a strong focus. A good infrastructure helps to plan, tailor and conduct clinical trials in a way they are easy and quickly analyzable.

In this research topic, we want to discuss new ideas for intelligent trial designs and concepts for data management.

## Author Contributions

All authors wrote and revised the editorial.

## Conflict of Interest Statement

The authors declare that the research was conducted in the absence of any commercial or financial relationships that could be construed as a potential conflict of interest.

